# Deep learning for glioblastoma segmentation using preoperative magnetic resonance imaging identifies volumetric features associated with survival

**DOI:** 10.1007/s00701-020-04483-7

**Published:** 2020-07-13

**Authors:** Yizhou Wan, Roushanak Rahmat, Stephen J. Price

**Affiliations:** Division of Neurosurgery, Department of Clinical Neurosciences, University of Cambridge, Level 3 A Block Box 165, Cambridge Biomedical Campus, Cambridge, CB2 0QQ UK

**Keywords:** Glioblastoma, Volumetric, Segmentation, MRI, Survival, Deep learning

## Abstract

**Background:**

Measurement of volumetric features is challenging in glioblastoma. We investigate whether volumetric features derived from preoperative MRI using a convolutional neural network–assisted segmentation is correlated with survival.

**Methods:**

Preoperative MRI of 120 patients were scored using Visually Accessible Rembrandt Images (VASARI) features. We trained and tested a multilayer, multi-scale convolutional neural network on multimodal brain tumour segmentation challenge (BRATS) data, prior to testing on our dataset. The automated labels were manually edited to generate ground truth segmentations. Network performance for our data and BRATS data was compared. Multivariable Cox regression analysis corrected for multiple testing using the false discovery rate was performed to correlate clinical and imaging variables with overall survival.

**Results:**

Median Dice coefficients in our sample were (1) whole tumour 0.94 (IQR, 0.82–0.98) compared to 0.91 (IQR, 0.83–0.94 *p* = 0.012), (2) FLAIR region 0.84 (IQR, 0.63–0.95) compared to 0.81 (IQR, 0.69–0.8 *p* = 0.170), (3) contrast-enhancing region 0.91 (IQR, 0.74–0.98) compared to 0.83 (IQR, 0.78–0.89 *p* = 0.003) and (4) necrosis region were 0.82 (IQR, 0.47–0.97) compared to 0.67 (IQR, 0.42–0.81 *p* = 0.005). Contrast-enhancing region/tumour core ratio (HR 4.73 [95% CI, 1.67–13.40], corrected *p* = 0.017) and necrotic core/tumour core ratio (HR 8.13 [95% CI, 2.06–32.12], corrected *p* = 0.011) were independently associated with overall survival.

**Conclusion:**

Semi-automated segmentation of glioblastoma using a convolutional neural network trained on independent data is robust when applied to routine clinical data. The segmented volumes have prognostic significance.

**Electronic supplementary material:**

The online version of this article (10.1007/s00701-020-04483-7) contains supplementary material, which is available to authorized users.

## Introduction

The median survival for glioblastoma remains approximately 12–15 months despite surgery and chemoradiation [1]. Extent of resection (EOR) is the only modifiable prognostic factor [1–3]. Other prognostic variables such as age, O^6^-methylguanine–DNA methyltransferase (*MGMT*) methylation and Karnofsky performance status (KPS) are not modifiable, and mutation status cannot be assessed preoperatively [1, 4]. Combining clinical variables with MRI features may be able to better prognosticate patients and stratify them for clinical trials—especially trials at the time of surgery when molecular markers are unknown [5, 6].

Radiogenomic analysis has linked MRI-based tumour subregions such as fluid-attenuated inversion recovery (FLAIR) volumes with genetic signatures of invasiveness and reduced survival [7]. Reproducible and accurate descriptors of imaging features are needed to identify prognostic biomarkers [8]. The Visually Accessible Rembrandt Images (VASARI) variables is the largest standardised dataset of imaging variables based on preoperative MRI [8, 9]. Qualitative VASARI descriptors when combined with manually segmented volumetric variables are independently associated with survival [9].

Manual segmentation of tumour volumes is time-consuming and suffers from high interobserver and intraobserver variability [10]. This limits their use in clinical practice. Computer-assisted methods reduce segmentation time and demonstrate good agreement with manual ground truth segmentations [10–12].

Convolutional neural networks (CNNs) are the state-of-the art computer-assisted method for glioblastoma segmentation [13]. They outperform alternative methods which use independent decision classifiers to extract texture and intensity features [14]. A pre-annotated dataset is used to train the CNN architecture to perform a series of mathematical convolutions through interdependent multiple layers. This determines the relationship between the input images and output images. The CNN can then be validated on different test datasets.

DeepMedic is a 3-dimensional (3D) CNN ranked highly in the Brain Tumour Segmentation Challenge (BRATS) [14]. DeepMedic assigns classes to each voxel independently using intensity and local feature information across image planes through two 11-layer convolutional pathways [14]. Each pathway samples different resolutions to lower computational cost [14]. DeepMedic has been shown to have robust segmentation accuracy of tumour subregions when tested and trained on MRI images performed at multiple institutions, with different protocols [15].

Automated segmentation of glioblastoma subregions has moderate agreement with their corresponding VASARI-derived semi-quantitative measures. However, measurement of certain tumour subregions is less accurate than other regions such as necrosis compared to the contrast-enhancing region (CER) [8]. In clinical practice, manual correction of segmentations generated from deep learning is more accurate than when the automated segmentations are used as full replacement for manual segmentations [15]. Generating curated training data for each dataset is also labour-intensive and time-consuming, requiring manual segmentations for images in each data cohort. Using publicly available data such as BRATS to train CNNs may enable these automated methods to be applicable across different datasets and institutions. We therefore aim to investigate whether DeepMedic trained on BRATS can be used for transfer learning, utilising automated segmentations as priors for manual correction. Previous studies have not examined the prognostic value of CNN-assisted volumetric measurements in combination with known prognostic variables. We test the validity of our semi-automated segmentation approach by correlating volumetric features of the resulting segmentations with survival.

## Methods

### Patient characteristics

All patients (≥ 18 years) diagnosed with histologically confirmed primary glioblastoma were identified from July 2016 to January 2018. Patients with previous glioma or cranial surgery were excluded.

Clinical characteristics were collected from electronic patient records. Motor deficit was defined as reduced power in any modality, and sensory deficit as reduced sensation or paraesthesia in any modality. Speech problems can present as receptive or expressive dysphasia. The operative records were used to obtain the following factors: American Association of Anesthesiologists (ASA) grade, use of 5-aminoleuvenic acid (5-ALA) and/or neurostimulation/awake surgery. Postoperative neurological deficit was recorded within 1 week from surgery.

Presence of isocitrate dehydrogenase (*IDH)* mutation and *MGMT* promoter methylation were recorded. *MGMT* promoter methylation was determined by pyrosequencing of the differentially methylated region 2 using a 10% cutoff value [16].

Patients were treated with either adjuvant chemoradiation (Stupp regimen), radiotherapy for symptom stabilisation or supportive care. The date of death was obtained from national patient records. The date of last follow-up was the time of query of NHS Spine (22/01/2019).

Patient demographics and imaging were anonymised prior to recording of research data. This study was approved by the local research ethics committee (Study ID: PRE.2017.040).

### Image preprocessing

MRI protocols are shown in Supplementary Table A1. Each patient’s images were resampled to 1 mm^3^. T1-weighted, T2-weighted and T2-FLAIR images were co-registered to T1C image using the FSL linear image registration tool (FLIRT) with a mutual information algorithm and 6-degrees of freedom [17].

Brain extraction was performed using brain extraction tool (BET) for the T1C image [18]. Masks were edited and applied to the other sequences using voxel multiplication.

Processed images were registered to the same atlas (SRI24) used for BRATS with a 12 degree of freedom affine registration, mutual information algorithm with FSL FLIRT [17]. Noise reduction was performed using smallest univalue segment assimilating nucleus (SUSAN) [19]. Images were denoised and normalised to zero mean unit variance.

### Tumour segmentation

The BRATS 2017 dataset contains 285 mixed-grade gliomas with expert-annotated manual segmentations of the (1) necrotic core (NC), (2) CER, (3) non-enhancing tumour (NET) and (4) peritumoural oedema (PTE) [20]. The definitions for manual segmentation are found in the Supplementary Data. DeepMedic can detect three tumour subregions: (1) FLAIR region, (2) CER and (3) NC region. Following the procedure from BRATS, automated segmentations of the whole-tumour (WT) region were created from T2-weighted sequences and FLAIR sequences whilst the CER region and NC regions were created from T1-weighted images. PTE and NET were manually delineated from the FLAIR region [15].

The primary architecture of DeepMedic consists of two main parallel pathways, each consists of four feature extraction layers with 53 kernels for feature extraction, as well as two fully connected layers and one final classification layer. The multi-scale processing of different input channels is handled using the dual pathway to achieve a large receptive field for the final classification, whilst the cost computation remains low. The first pathway operates on the original image, and the second one operates on a down-sampled version [14, 21].

**Fig. 1:**
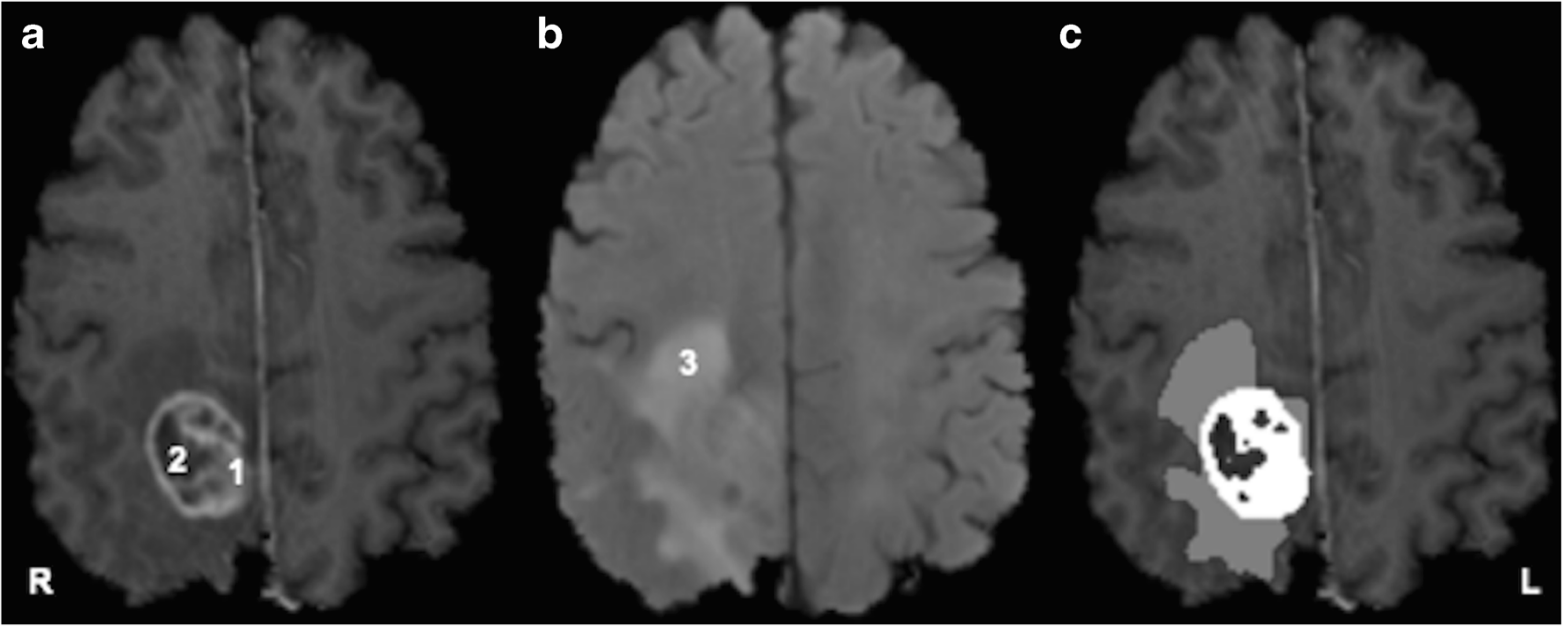
Example segmentation showing tumour subregions and imaging sequences. **a** T1-post-contrast. **b** T2 fluid-attenuated inversion recovery (FLAIR). **c** Whole-tumour mask. 1 = contrast-enhancing region = blue; 2 = necrotic core; 3 = peritumoural oedema. R = right. L = left

To apply transfer learning on our dataset, DeepMedic was firstly trained on randomly chosen scans from BRATS dataset. The dataset was divided into 107 patients for training (*n* = 66,340 images) and 50 patients for validation (*n* = 31,000 images). This trained model was further tested on 50 further patients from BRATS prior to application to our test dataset. As we are evaluating the validity of applying a CNN trained on a different dataset to our test dataset, one author (YW) manually segmented our test images by correcting the automated segmentation labels derived from DeepMedic using 3D Slicer (Harvard Medical School) (Fig. 1) [22].

DeepMedic incorporates data augmentation to increase input data volumes as well as their complexity via reflection with respect to the mid-sagittal plane. Data shuffle is performed at the start of each epoch to avoid overfitting [23]. The hyper parameters remained the same as the original DeepMedic network proposed [14]. The network is regularised using dropout, 35 epochs and 5 batch sizes, with 5-fold cross-validation. The loss function used was negative log-likelihood.

Training was performed using an implementation of DeepMedic on Tensorflow, using an NVIDIA Titan Xp graphics card [24].

**Fig. 2:**
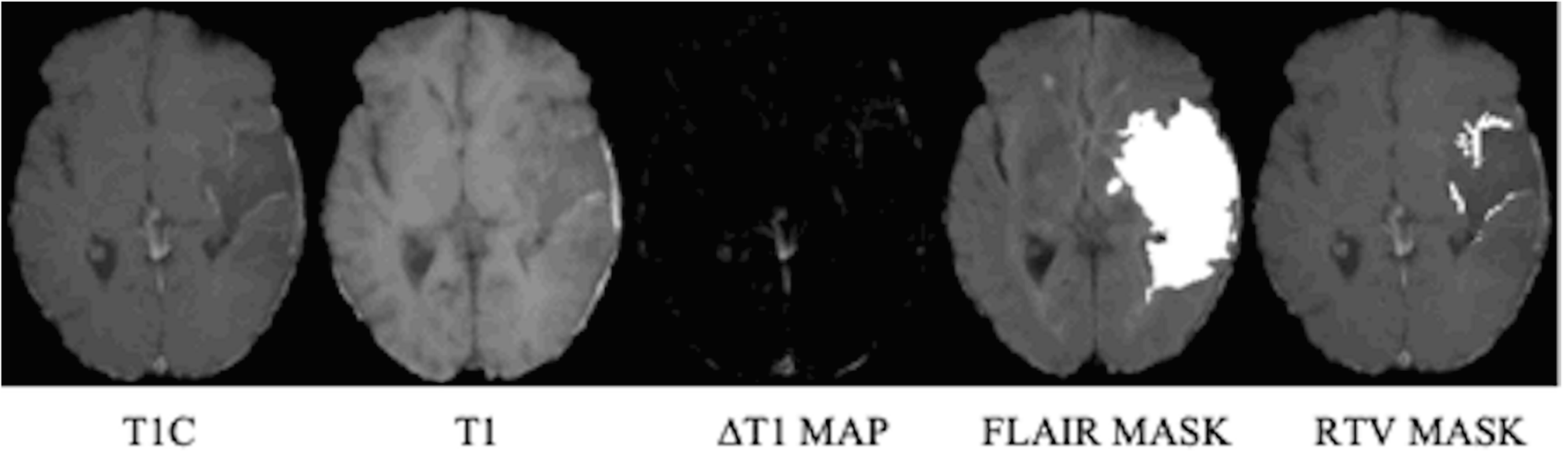
Processing pipeline for segmenting residual enhancing tumour (radiological orientation). T1C = T1 contrast; T2 FLAIR = T2 fluid-attenuated inversion recovery; RTV = residue tumour volume

The delimitation of residue contrast-enhancing tumour was performed using T1 subtraction maps (ΔT1 map). This method improves the delineation of tumour by subtraction of contrast enhancement from blood products [25]. Their use has validated residue tumour volume (RTV) as a predictor of survival [25]. ΔT1 maps were created by voxel-by-voxel subtraction of the pre-processed T1 image from the T1C image (Fig. 2).

The comparative metrics used to assess the quality of the segmentations were the Dice coefficient and volumes of the segmentations. The Dice coefficient is a value between zero and one which presents the degree of overlap between two segmentations (one represents perfect overlap) [11]. For our test dataset, the post-manually edited tumour subregion segmentations were considered the ground truth for comparison with the automated labels. Differences between the automated and manually corrected segmentations volumes of each tumour subregion were also compared. For the BRATS test data (*n* = 50), we also computed the Dice coefficient between the automated labels generated from our model and the expert-annotated manual ground truths.

### Statistical analysis

VASARI features were scored on preoperative imaging (Supplementary Table A2). Spearman rank correlation was used to compare the proportions of each subregion; EOR was calculated based on volumetric segmentations; EOR = CER − RTV/CER × 100%.

The primary outcome was overall survival (OS): difference between the date of death and the date of surgery. Cox proportional hazards models were used to identify significant factors associated with OS. Median follow-up time was calculated using the reverse KM method [26]. The Student *t* test and Wilcoxon rank-sum test were used to compare baseline characteristics between groups for continuous variables. The chi-square test was used for comparing categorical variables.

Variables with *p* < 0.2 in the bivariable regression models were included in the multivariable models. The Akaike information criterion (AIC) was used as a measure to compare the quality of the models. AIC scores compare the relative performance of a model based on the number of parameters and goodness of fit. The model with the lower AIC score explains the greatest variation using the least number of independent variables [27]. Multiple testing was controlled for using the false discovery rate. A false discovery rate adjusted *p* value (*q*-value) is the percentage of significant tests which will result in a false positive. Given the exploratory nature of the study, a threshold of 0.1 was chosen (sensitivity analysis showed that lowering the threshold to 0.05 did not change the results of significant variables). Statistical analysis used Stata version 14 (StataCorp. College Station, Texas).

## Results

### Patient, clinical and treatment characteristics

One hundred twenty cases were included (Table 1). Median follow-up time was 19.9 months (95% CI, 17.4–21.9 months). The median OS was 8.8 months (95% CI, 7.3–12.0 months). Median survival of patients undergoing resection was 8 months longer than patients undergoing biopsy (12.8 months vs 4.7 months, *p <* 0.001).

**Table 1:** Baseline characteristics of all patients (*n* = 120)

Characteristics	Value
Age (years)^*a*^	65.2 [57.1–70.6]
Male (%)	69 (57.5)
KPS (%)	
< 70	2 (1.7)
70	5 (4.2)
80	20 (16.7)
90	60 (50.0)
100	33 (27.5)
Presenting symptoms (%)	
Headache	44 (36.7)
Seizure	38 (31.7)
Confusion/memory	51 (42.5)
Vision	24 (20)
Language	45 (37.5)
Motor	46 (38.3)
Sensory	18 (15)
Perioperative variables (%)	
Steroid use	108 (90)
ASA grade	
1	8 (6.7)
2	86 (71.7)
3	25 (20.8)
4	1 (0.8)
Preoperative deficit	117 (97.5)
Postoperative deficit (*n* = 75)	
None	54 (45.0)
New/worsened	29 (24.2)
Improved	37 (30.8)
Surgical variables (%)	
Awake	15 (12.5)
Intraoperative stimulation	33 (27.7)
Biopsy	45 (37.5)
CRET	59 (49.2)
PRET	16 (13.3)
EOR (%) [range]^*b*^	99 [50.9–100]
RTV (cm^3^) [IQR]^*c*^	0.47 [0.09–0.73]
Tumour variables (%)	
*IDH* mutant	4 (3.3)
*MGMT* methylated	52 (43.3)
Adjuvant therapy (%)	
Chemoradiotherapy	76 (63.3)
Radiotherapy	27 (22.5)
None	17 (14.2)
Median OS (months) (95% CI)^*d*^	8.8 (7.3–12.0)
Alive at last follow-up (%)	25 (20.8)

*ASA* = American Association of Anesthesiologists; *CI* = confidence interval; *CRET* = complete resection of enhancing tumour; *EOR* = extent of resection; *IDH* = isocitrate dehydrogenase; *IQR* = interquartile range; *KPS* = Karnofsky Performance Status; *PRET* = partial resection of enhancing tumour; *RTV* = residual tumour volume; *MGMT* = O^6^-methylguanine–DNA methyltransferase. ^*a*^Median [interquartile range]. ^*b*^Median [interquartile range]. ^*c*^Based on number of patients who underwent resection with residual contrast enhancement on postoperative MRI. ^*d*^Log-rank test of equality of survivor function

The median age was 65.2 [57.1–70.6] years and 69 (57.5%) of patients were male. Complete resection of enhancing tumour (CRET) was achieved in 59 (49.2%) patients and partial resection of enhancing tumour (PRET) achieved in 16 (13.3%) patients. A biopsy was performed on 45 (37.5%) patients. The median EOR was high, 99% (IQR, 50.9–100%) with a median RTV of 0.47 cm^3^ (IQR, 0.09–0.73 cm^3^) for PRET tumours.

Due to differences between baseline characteristics, the resection and biopsy groups were analysed separately. Resection patients had larger CER and NC volumes whilst biopsy patients had a larger NET volume (Supplementary Table B1).

### Comparison between automated and manually corrected segmentations

The network was able to detect WT, CER and NC in all patients in the BRATS test data and in 118 patients (98%) of our test dataset. Failure to localise the tumour was caused by inaccurate labelling due to T2 hyperintensities on the FLAIR sequence in two patients. The time taken for full manual segmentation in each of these two patients was approximately 40 min. Time taken for manual correction of the automated labels in the remaining patients was approximately 15 min. The training and test times were approximately 41 h (2475 min) and 36 min respectively in our data. Therefore, considering training, testing and correction time, our semi-automated segmentation method can reduce segmentation time by approximately 38 h (2289 min) compared to manual segmentation.

**Fig. 3:**
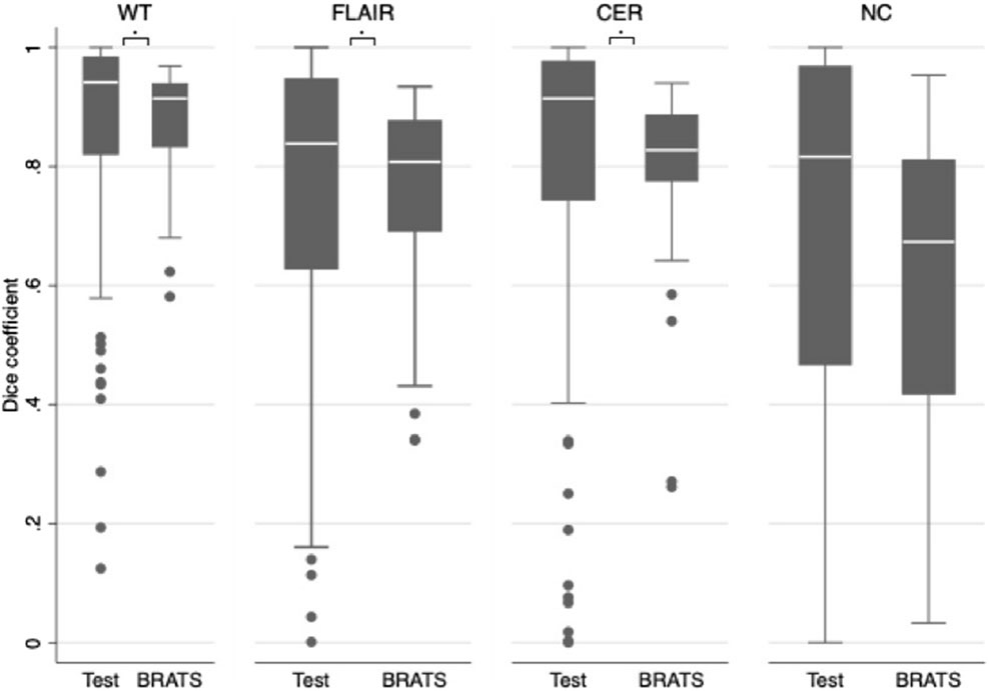
Box and whisker plots (IQR) of WT, FLAIR, CER and NC regions Dice coefficients between the BRATS test data (BRATS) and sample data (Test). CER = contrast-enhancing region; FLAIR = fluid-attenuated inversion recovery; NC = necrotic core; WT = whole tumour

Comparison of segmentation outcomes between our BRATS test data (*n* = 50) and our sample data (*n* = 120) showed similar network performance in the two datasets. Median Dice coefficients in our sample were (1) WT 0.94 (IQR, 0.82–0.98), (2) FLAIR region 0.84 (IQR, 0.63–0.95) and (3) CER 0.91 (IQR, 0.74–0.98) and NC were 0.82 (IQR, 0.47–0.97). We observed significantly different Dice coefficients for WT 0.91 (IQR, 0.83–0.94 *p* = 0.012), CER 0.83 (IQR, 0.78–0.89 *p* = 0.003) and NC 0.67 (IQR, 0.42–0.81 *p* = 0.005) but not FLAIR region 0.81 (IQR, 0.69–0.8 *p* = 0.170) in the BRATS test data (Fig. 3).

**Fig. 4:**
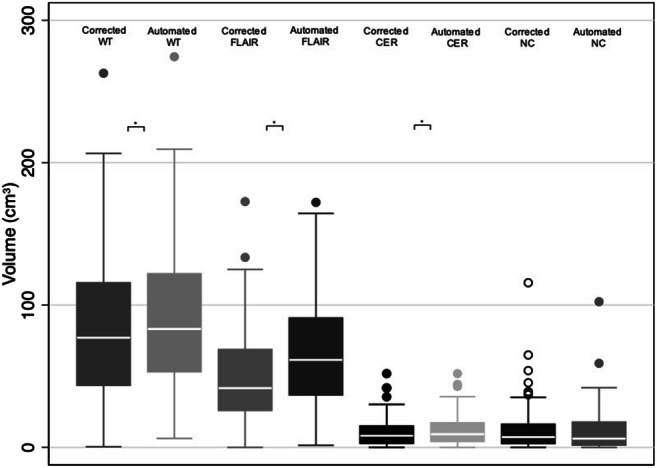
Box and whisker plots (IQR) comparing tumour subregion volume between corrected and automated segmentations for WT, FLAIR, CER and NC regions. CER = contrast-enhancing region; FLAIR = fluid-attenuated inversion recovery; NC = necrotic core; WT = whole tumour

The median tumour volume for post-corrected segmentations were WT 77.0 cm^3^ (IQR, 43.6–115.6), FLAIR region 41.5 cm^3^ (IQR, 26.0–68.7), CER 8.2 cm^3^ (IQR, 2.9–14.9) and NC 7.2 cm^3^ (IQR, 2.7–16.2). There were significant differences between automated segmentation volumes for WT 83.1 cm^3^ (IQR, 53.1–121.9 *p <* 0.001), FLAIR region 61.5 cm^3^ (IQR, 36.8–90.9 *p <* 0.001) and CER 9.3 cm^3^ (IQR, 4.1–17.3 *p <* 0.001) but not NC 6.2 cm^3^ (IQR, 1.6–17.7 *p =* 0.209) (Fig. 4).

### Relationship between volumetric subregions

**Fig. 5:**
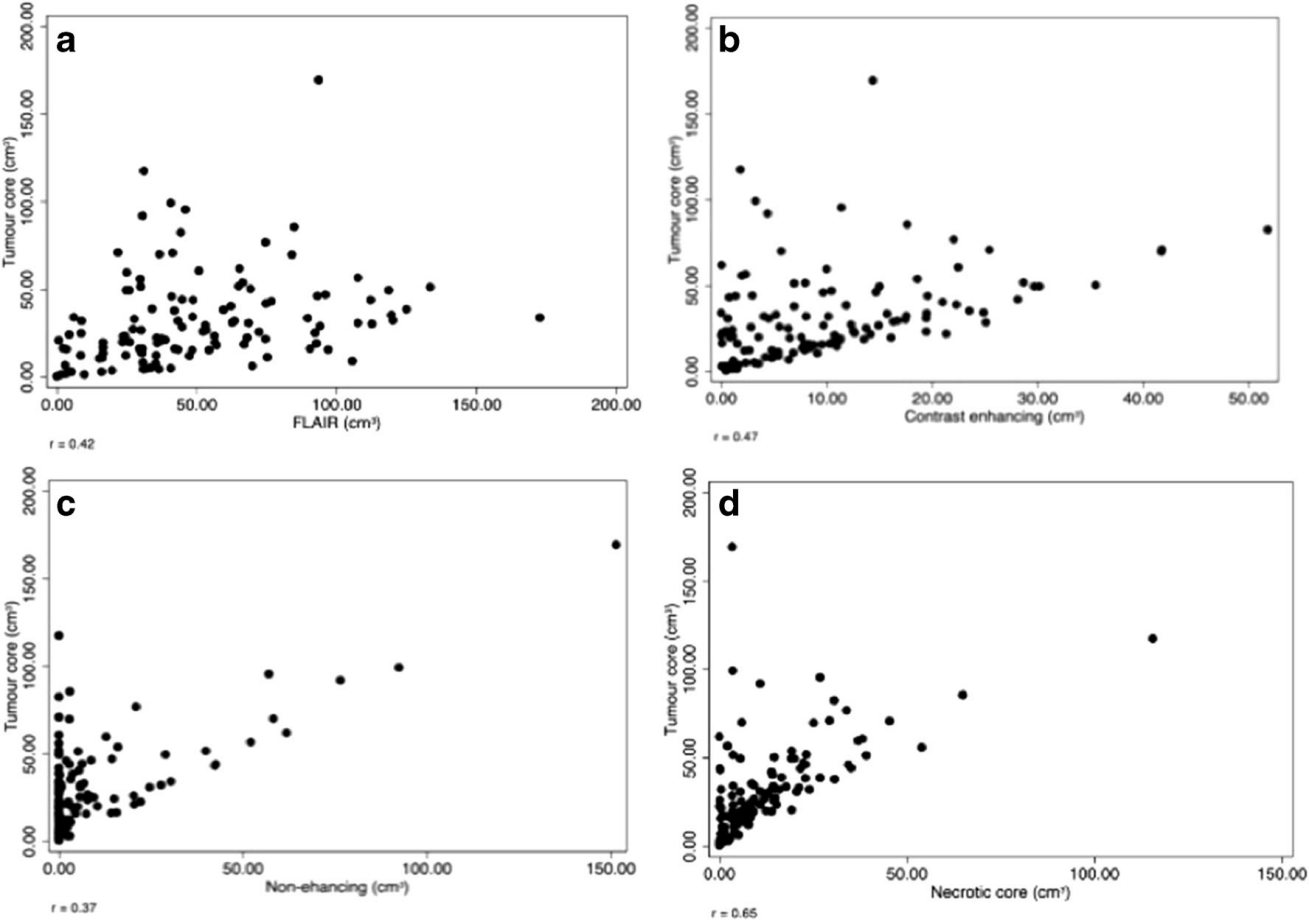
Scatterplots showing correlation between tumour subregions and tumour core volume. The correlation was strongest for contrast enhancing and necrosis (*r* = 0.65) volume (*r* = 0.42) (**b** and **d**) whilst there was a weaker correlation between oedema (*r* = 0.37) and non-enhancing (*r* = 0.36) volume (**a** and **c**)

There was a positive correlation between the volume of tumour core and individual tumour subregions (*p* < 0.001), shown in Fig. 5.

PTE volume positively correlated with CER volume *r* = 0.42 (*p* < 0.001) and NC volume *r* = 0.37 (*p* < 0.001) but not NET volume *r* = 0.06 (*p* = 0.508). There was a negative correlation between CER volume and NET volume *r* = − 0.31 (*p* < 0.001) as well as between CER volume and NC volume *r* = 0.37 (*p* < 0.001). NET volume was not correlated with NC volume *r* = −0.107 (*p* = 0.247).

Subregion volumes were normalised by dividing by TC volume. TC volume positively correlated with NET/TC *r* = 0.22 (*p* < 0.05). There was a negative correlation between TC and CER/TC *r* = − 0.35 (*p* < 0.001) and TC with PTE/TC *r* = − 0.48 (*p* < 0.001). NC/TC did not correlate with TC volume *r* = 0.13 (*p* = 0.158).

PTE/NC ratio correlated with CER/TC *r* = 0.27 (*p =* 0.003) and was independent of NET/TC *r* = 0.10 (*p =* 0.285). NER/TC was independent of CER/TC *r* = − 0.16 (*p* = 0.080) and negatively correlated with NET/TC *r* = − 0.49 (*p* < 0.001).

### Volumetric features associated with overall survival

Cox regression models were constructed for volumetric variables which were not significantly correlated with each other (Supplementary Table C1, C3 and C5). Analysis was performed for the entire cohort and for biopsy and resection patients separately (Table 2).

**Table 2:** Cox regression analysis

Variable	Univariable HR (95% CI)								Multivariable HR^*a*^ (95% CI)						
	All patients	*p*	Biopsy	*p*	Resection	*p*	All patients^*b*^	*p*	*q*	Biopsy^*c*^	*p*	*q*	Resection^*d*^	*p*	*q*
Clinical variables															
Age	1.04 (0.28–7.67)	< 0.001	1.00 (0.97–1.03)	0.997	1.05 (1.03–1.08)	< 0.001	1.05 (1.02–1.08)	*0.003*	*0.014*	1.04 (0.98–1.11)	0.179	–	1.08 (1.03–1.14)	*0.002*	**–**
Sex (female)	Reference	–	–	–	–	–	–	–	–	–	–	–	–	–	–
Male	1.28 (0.85–1.93)	0.240	1.18 (0.64–2.17)	0.593	1.61 (0.91–2.84)	0.099	–	–	–	–	–	–	1.38 (0.66–2.90)	0.390	–
-KPS < 70	Reference	–	–	–	–	–	–	–	–	–	–	–	–	–	–
70	1.47 (0.21–1.00)	0.645	4.62 (0.47–45.89)	0.191	0.07 (0.04–1.05)	0.054	5.14 (0.76–34.6)	0.094	–	2024.14 (15.32–267,501.6)	*0.002*	*0.012*	0.04 (0.006–2.59)	0.131	–
80	0.90 (0.21–3.90)	0.890	3.08 (0.40–23.82)	0.280	0.02 (0.01–0.24)	0.002	1.12 (0.18–6.88)	0.901	–	44.39 (0.82–2404.33)	0.063	–	0.09 (0.002–0.416)	*0.016*	**–**
90	0.67 (0.16–2.76)	0.578	2.51 (0.33–19.20)	0.376	0.03 (0.03–0.36)	0.005	1.05 (0.20–5.61)	0.954	–	96.31 (1.83–5069.28)	*0.024*	–	0.01 (0.003–0.450)	*0.017*	**–**
100	0.36 (0.08–1.58)	0.178	1.38 (0.16–11.94)	0.767	0.02 (0.00–0.21)	0.001	0.78 (0.14–4.36)	0.774	–	9.37 (0.28–318.65)	0.214	–	0.01 (0.003–0.343)	*0.011*	**–**
Headache	0.78 (0.51–1.19)	0.243	0.82 (0.42–1.60)	0.565	0.83 (0.47–1.45)	0.505	–	–	–	–	–	–	–	–	–
Seizure	0.76 (0.49–1.19)	0.234	0.47 (0.24–0.90)	0.022	0.74 (0.40–1.39)	0.351	–	–	–	1.45(0.28–7.43)	0.659	–	–	–	–
Cognitive	1.71 (1.14–2.58)	0.010	1.17 (0.64–2.16)	0.605	2.16 (1.24–3.76)	0.007	1.31 (0.78–2.19)	0.304	–	–	–	–	1.84 (0.66–5.14)	0.244	–
Vision	1.37 (0.84–2.22)	0.209	1.57 (0.55–4.47)	0.394	1.99 (1.11–3.55)	0.020	–	–	–	–	–	–	1.79 (0.55–5.85)	0.333	–
Language	1.26 (0.83–1.90)	0.275	0.786 (0.43–1.45)	0.442	1.40 (0.80–2.46)	0.235	–	–	–	–	–	–	–	–	–
Motor	0.91 (0.60–1.39)	0.674	1.04 (0.56–1.95)	0.895	0.79 (0.45–1.39)	0.417	–	–	–	–	–	–	–	–	–
Sensory	0.96 (0.53–1.72)	0.879	0.74 (0.36–1.51)	0.405	0.56 (0.17–1.79)	0.325	–	–	–	–	–	–	–	–	–
Steroid use	1.26 (0.63–2.50)	0.512	1.98 (0.87–4.52)	0.103	1.95 (0.47–8.08)	0.356	–	–	–	6.96 (1.02–47.49)	*0.048*	–	–	–	–
Preoperative deficit	2.61 (0.64–10.67)	0.180	–	–	2.09 (0.50–8.66)	0.309	0.78 (0.12–5.04)	0.790	–	–	–	–	–	–	–
ASA grade 1 (Reference)	–	–	–	–	–	–	–	–	–	–	–	–	–	–	–
2	2.91 (1.05–8.08)	0.04	2.32 (0.82–6.59)	0.113	2.91 (1.05–8.08)	0.04	0.75 (0.22–2.50)	0.639	–	0.47(0.12–1.78)	0.264	–	1.18 (0.27–5.17)	0.830	–
3	4.02 (1.35–11.99)	0.012	2.44 (0.73–8.16)	0.148	4.02 (1.35–11.99)	0.012	2.09 (0.55–8.00)	0.282	–	–	–	–	2.39 (0.37–15.20)	0.357	–
4	34.36 (3.50–337.69)	0.002	161.52 (8.52–3061.44)	0.001	34.36(3.50–337.69)	0.002	4.48 (0.28–71.79)	0.290	–	–	–	–	81.11(0.80–8252.08)	0.062	–
Surgery (Biopsy)	Reference	–	–	–	–	–	–	–	–	–	–	–	–	–	–
CRET	0.29 (0.18–0.45)	< 0.001	–	–	–	–	0.11 (0.03–0.38)	*< 0.001*	*0.006*	**–**	**–**	–	–	–	–
PRET	0.59 (0.31–1.10)	0.095	–	–	–	–	0.23 (0.06–0.93)	*0.039*	**–**	**–**	**–**	–	–	–	–
Awake	–	–	–	–	0.56 (0.27–1.15)	0.115	–	**–**	**–**	**–**	**–**	–	0.79 (0.19–3.32)	0.753	–
Stimulation	–	–	–	–	0.63 (0.36–1.10)	0.108	–	**–**	**–**	**–**	**–**	–	1.28 (0.48–3.40)	0.625	–
EOR	–	–	–	–	0.02 (5.53 × 10^−5^–0.09)	0.001	–	**–**	**–**	**–**	**–**	–	3.69 × 10^−4^ (1.51 × 10^−7^–0.90)	*0.047*	**–**
RTV	–	–	–	–	1.14 (1.03–1.25)	0.011	–	**–**	**–**	**–**	**–**	–	1.13 (0.94–1.36)	0.208	–
Postoperative deficit (none)	Reference	–	–	–	–	–	–	–		–	–	–	–	–	–
New/worsened	1.39 (0.85–2.30)	0.191	1.30 (0.62–2.73)	0.482	1.52 (0.77–2.99)	0.227	3.42 (1.69–6.90)	*0.001*	*0.008*	3.37 (0.80–14.18)	0.098	**–**	**–**	**–**	**–**
Improved	0.68 (0.42–1.10)	0.113	0.50 (0.23–1.07)	0.075	0.77 (0.41–1.44)	0.410	0.60 (0.32–1.14)	0.121	–	0.09(0.02–0.48)	*0.005*	*0.021*	–	–	–
*IDH* mutant	0.14 (0.02–1.02)	0.052	–	–	0.18 (0.02–1.28)	0.086	0.49 (0.04–5.71)	0.571	–	–	–	–	–	–	–
*-MGMT* methylated	0.92 (0.61–1.38)	0.688	0.70 (0.37–1.36)	0.294	1.11 (0.64–1.90)	0.716	–	–	–	–	–	–	–	–	–
Adjuvant (none)	Reference	–	–	–	–	–	–	–	–	–	–	–	–	–	–
Radiation	0.63 (0.34–1.17)	0.142	0.66 (0.29–1.54)	0.341	0.59 (0.23–1.52)	0.277	0.46 (0.19–1.11)	0.085	–	0.23(0.05–0.98)	*0.047*	–	–	–	–
Chemoradiation	0.16 (0.09–0.29)	< 0.001	0.27 (0.12–0.63)	0.002	0.13 (0.06–0.30)	< 0.001	0.15 (0.06–0.33)	*< 0.001*	*0.003*	0.05(0.01–0.29)	*0.001*	*0.004*	0.15 (0.03–0.85)	*0.031*	**–**
VASARI variables															
Side (right)	Reference	–					–	–	–	–	–	–	–	–	–
Left	1.70 (1.11–2.63)	0.016	1.02 (0.52–2.00)	0.964	1.95 (1.11–3.43)	0.021	1.18 (0.63–2.19)	0.607	–	–	–	–	1.16 (0.51–2.68)	0.720	–
Bilateral	2.07 (0.28–15.28)	0.477	0.64 (0.08–4.92)	0.663	–	–	0.56 (0.03–10.05)	0.696	–	–	–	–	–	–	–
Lobe (frontal)	Reference	–	–	–	–	–	–	–	–	–	–	–	–	–	–
Temporal	0.88 (0.54–1.44)	0.612	0.50 (0.22–1.13)	0.095	1.03 (0.55–1.93)	0.934	0.79 (0.38–1.63)	0.525	–	0.95 (0.12–7.32)	0.959	–	0.75 (0.28–2.01)	0.563	–
Insula	0.81 (0.19–3.42)	0.778	0.24 (0.05–1.11)	0.069	–	–	0.16 (0.02–1.20)	0.075	–	0.12 (0.0005–0.30)	*0.007*	*0.029*	–	–	–
Parietal	0.96 (0.53–1.73)	0.890	0.53 (0.22–1.39)	0.211	0.98 (0.44–2.18)	0.963	1.03 (0.40–2.63)	0.951	–	0.66(0.03–14.08)	0.790	–	0.77 (0.16–3.64)	0.743	–
Occipital	1.98 (0.68–5.76)	0.211	0.53 (0.07–4.17)	0.548	3.91 (1.09–14.07)	0.037	10.05 (1.27–79.66)	*0.029*	*0.022*	78.90 (0.87–7181.20)	0.058	–	5.14 (0.49–53.78)	0.171	–
Brainstem	8.86 (1.13–69.50)	0.038	2.77(0.33–22.95)	0.344	–	–	4.54 (0.39–53.44)	0.229	–	741.00 (14.34–3890.97)	*0.001*	*0.008*	–	–	–
Callosum	2.71 (0.93–7.88)	0.067	0.72(0.23–2.27)	0.571	–	–	1.38 (0.23–8.34)	0.729	–	2.15(0.36–12.70)	0.399	–	–	–	–
Eloquent (none)	Reference	–	–	–	–	–	–	–	–	–	–	–	–	–	–
Speech motor	1.15 (0.60–2.24)	0.662	0.69(0.21–2.22)	0.531	1.06 (0.47–2.39)	0.896	2.63 (1.02–6.75)	*0.045*	*0.019*	22.28 (1.83–271.11)	*0.015*	*0.033*	2.62 (0.79–8.65)	0.114	–
Speech receptive	1.31 (0.68–2.52)	0.427	0.73(0.27–1.96)	0.535	0.82 (0.29–2.31)	0.708	0.87 (0.35–2.14)	0.757	–	2.09(0.24–18.41)	0.508	–	3.09 (0.70–13.72)	0.138	–
Motor	0.92 (0.47–1.77)	0.793	0.58(0.23–1.52)	0.269	0.41 (0.13–1.34)	0.142	0.42 (0.15–1.17)	0.098	–	0.13(0.03–0.53)	*0.005*	*0.017*	0.49 (0.09–2.61)	0.399	–
Vision	1.61 (0.91–2.85)	0.101	0.41(0.17–0.99)	0.047	3.58 (1.05–12.20)	0.042	0.18 (0.05–0.61)	*0.006*	–	0.17(0.01–3.18)	0.238	–	–	–	–
SVZ involvement	1.62 (1.08–2.43)	0.023	0.99 (0.44–2.25)	0.981	0.81 (0.42–1.58)	0.546	1.08 (0.49–2.38)	0.841	–	–	–	–	–	–	–
Cortical involvement	0.69 (0.25–1.87)	0.461	0.90(0.27–2.92)	0.854	1.17 (0.16–8.53)	0.875	–	–	–	–	–	–	–	–	–
White matter involvement	1.79 (1.17–2.73)	0.007	1.25 (0.64–2.45)	0.519	0.66 (0.26–1.67)	0.381	1.77 (0.75–4.22)	0.195	–	–	–	–	–	–	–
NER crossing	2.07 (1.11–3.85)	0.021	1.15(0.55–2.40)	0.701	1.31 (0.32–5.39)	0.713	0.74 (0.28–1.98)	0.548	–	–	–	–	–	–	–
CER crossing	2.00 (0.92–4.34)	0.081	1.56 (0.68–3.58)	0.292	–	–	0.63 (0.18–2.23)	0.476	–	–	–	–	–	–	–
Multifocal	2.95 (1.65–5.28)	< 0.001	1.61 (0.78–3.32)	0.201	2.74 (0.98–7.70)	0.055	2.28 (0.75–6.98)	0.148	–	–	–	–	1.30 (0.09–18.50)	0.846	–
Volumetric variables															
FLAIR volume	1.00 (0.99–1.00)	0.183	1.00(0.99–1.00)	0.501	1.00 (0.99–1.00)	0.209	1.00 (0.99–1.00)	0.290	–	–	–	–	–	–	–
WT volume	1.00 (0.99–1.00)	0.210	1.00(0.99–1.00)	0.946	1.00 (0.99–1.00)	0.309	–	**–**	–	–	–	–	–	**–**	**–**
TC volume	1.00 (0.99–1.00)	0.385	1.00(0.98–1.01)	0.663	1.00 (0.99–1.01)	0.366	–	**–**	–	–	–	–	–	**–**	**–**
PTE volume	1.00 (0.99–1.00)	0.279	1.00(0.99–1.01)	0.635	1.00 (0.99–1.01)	0.497	–	**–**	–	–	–	–	–	**–**	**–**
CER volume	1.01 (0.99–1.03)	0.448	1.02(0.98–1.06)	0.292	1.03 (1.00–1.05)	0.061	–	**–**	–	–	–	–	0.99 (0.94–1.05)	0.760	–
NET volume	1.00 (0.99–1.00)	0.384	0.99(0.97–1.00)	0.096	0.98 (0.96–1.01)	0.218	–	**–**	–	0.01 (0.002–0.23)	*0.005*	–	–	**–**	**–**
NC volume	1.00 (0.98–1.01)	0.525	1.03(1.01–1.06)	0.014	0.99 (0.98–1.01)	0.485	–	**–**	–	0.99(0.95–1.04)	0.759	–	–	**–**	**–**
FLAIR/TC ratio	0.94 (0.85–1.04)	0.255	1.02(0.89–1.18)	0.764	0.91 (0.79–1.04)	0.172	–	**–**	–	–	–	–	0.98 (0.79–1.22)	0.870	–
PTE/TC ratio	0.95 (0.86–1.04)	0.274	1.05(0.93–1.19)	0.440	0.91 (0.80–1.04)	0.177	–	**–**	–	–	–	–	0.98 (0.78–1.22)	0.844	–
NET/TC ratio	0.99 (0.52–1.87)	0.969	0.35(0.15–0.78)	0.011	1.09 (0.35–3.45)	0.881	–	**–**	–	0.09(0.07–1.20)	0.068	–	–	–	–
CER/TC ratio	2.06 (0.96–4.56)	0.075	1.98 (0.73–5.38)	0.179	4.27 (1.33–13.68)	0.014	4.73 (1.67232.12–13.40)	*0.003*	*0.017*	13.88 (1.56–123.36)	*0.018*	–	0.49 (0.10–2.41)	0.386	–
NC/TC ratio	0.46 (0.20–1.08)	0.074	5.33(1.65–17.26)	0.05	0.18 (0.05–0.62)	0.007	8.13 (2.06–32.12)	*0.003*	*0.011*	1.31(0.06–28.08)	0.865	–	0.85 (0.08–8.94)	0.895	–
FLAIR/WT ratio	0.54 (0.19–1.53)	0.243	0.23(0.06–0.86)	0.029	0.55 (0.13–2.38)	0.420	–	–	–	0.08 (0.001–4.57)	0.222	–	–	–	–
PTE/WT ratio	0.58 (0.22–1.54)	0.275	1.63(0.40–6.68)	0.68	0.57 (0.14–2.30)	0.431	–	–	–	–	–	–	–	–	–
CER/WT ratio	4.01 (1.02–15.76)	0.047	2.33(0.46–11.77)	0.307	10.45(1.36–80.42)	0.024	–	–	–	–	–	–	0.76 (0.19–31.11)	0.886	–
NET/WT ratio	1.01 (0.36–2.83)	0.980	0.20(0.05–0.84)	0.028	1.05 (0.16–7.09)	0.960	–	–	–	0.01 (0.002–0.23)	*0.005*	*0.025*	–	–	–
NC/WT ratio	0.84 (0.18–3.90)	0.283	12.47 (1.55–100.32)	0.018	0.38 (0.04–3.71)	0.407	–	–	–	1.83(0.06–60.85)	0.735	–	–	–	–
PTE/CER ratio	1.00 (1.00–1.00)	0.322	1.00 (1.00–1.00)	0.910	1.00 (0.99–1.01)	0.720	–	–	–	–	–	–	–	–	–
PTE/NET ratio	1.01 (1.00–1.01)	0.146	1.01 (1.00–1.02)	0.011	1.00 (0.99–1.02)	0.528	–	–	–	–	–	–	–	–	–
PTE/NC ratio	1.00 (1.00–1.00)	0.572	1.00 (1.00–1.00)	0.632	1.03 (1.00–1.05)	0.035	–	–	–	–	–	–	1.05 (1.01–1.09)	*0.02*	**–**
NET/NC ratio	1.00 (1.00–1.00)	0.445	1.00 (1.00–1.00)	0.868	1.01 (0.98–1.04)	0.447	–	–	–	–	–	–	–	–	–

*ASA* = American Association of Anesthesiologists; *CER* = contrast-enhancing region; *CRET* = complete resection of enhancing tumour; *EOR* = extent of resection; *FLAIR* = fluid-attenuated inversion recovery; *HR* = hazard ratio; *IDH* = isocitrate dehydrogenase; *KPS* = Karnofsky Performance Status; *MGMT* = O^6^-methylguanine–DNA methyltransferase; *NC* = necrotic core; *NET* = non-enhancing tumour; *PRET* = partial resection of enhancing tumour; *PTE* = peritumoural oedema; *RTV* = residual tumour volume; *SVZ* = subventricular zone; *TC* = tumour core; *WT* = whole tumour. ^*a*^Unless otherwise specified, the multivariable hazard ratio for the clinical variables is derived from the overall model with the lowest Akaike information criterion. Where a volumetric variable appears in more than one multivariable model, the hazard ratio displayed is derived from the model with the lowest Akaike information criterion (Appendix Tables C2, C4 and C6). ^*b*^Model 5 from Appendix Table C2. ^*c*^Model 8 from Appendix Table C4. ^*d*^Model 2 from Appendix Table C6

Significant p values are emphasised in italics

For the whole cohort, clinical variables associated with OS included the following: age (HR 1.05 [95% CI, 1.02–1.08], *p* = 0.003), postoperative deficit (HR 3.42 [95% CI, 1.69–6.90], *p* = 0.001), CRET (HR 0.11 [95% CI, 0.03–0.38], *p* < 0.001), PRET (HR 0.23 [95% CI, 0.06–0.93], *p* < 0.039) and adjuvant chemoradiotherapy (HR 0.15 [95% CI, 0.06–0.33], *p* < 0.001). Occipital lobe location (HR 10.05 [95% CI, 1.27–79.66]. *p* = 0.029), speech motor cortex location (HR 2.63 [95% CI, 1.02–6.75], *p* = 0.045) and visual cortex location (HR 0.18 [95% CI, 0.05–0.61], *p* = 0.006) were associated with OS. Volumetric variables which were significantly associated with OS were as follows: CER/TC (HR 4.73 [95% CI, 1.67–13.40], *p* = 0.003) and NC/TC (HR 8.13 [95% CI, 2.06–32.12], *p* = 0.003. At a corrected critical value of *q* = 0.022, both CER/TC (*q* = 0.017) and NC/TC (*q* = 0.011) were significantly associated with OS.

The significant variables associated with OS for resection patients were as follows: age (HR 1.08 [95% CI, 1.03–1.14), *p* = 0.002), KPS, EOR (HR 3.69 × 10^−4^ [95% CI, 1.51 × 10^−7^–0.90], *p* = 0.047) and adjuvant chemoradiotherapy (HR 0.15 [95% CI, 0.03–0.85), *p* = 0.031). The only significant volumetric feature was PTE/NC (HR 1.05 [95% CI, 1.01–1.09], *p* = 0.020) but this was not significant after adjustment for multiple testing (adjusted critical *q* > 0.004).

For biopsy patients, the following volumetric variables were associated with OS: NET volume (HR 0.01 [95% CI, 0.002–0.23], *p* = 0.005), NET/WT (HR 0.01 [95% CI, 0.002–0.23], *p* = 0.005) and CER/TC (HR 13.88 [95% CI, 1.56–123.36], *p* = 0.018). Only NET/WT was significantly associated with OS following correction for multiple testing (*q* < 0.038).

*MGMT* methylation and *IDH* mutation were not significantly associated with OS in bivariable or multivariable analysis.

## Discussion

In this study, we integrated a deep learning network into a clinically applicable processing pipeline for semi-automated measurement of glioblastoma volumetric features from preoperative MRI. A CNN was trained on publicly available BRATS data before testing on our routine clinical dataset. Final segmentation labels were generated by manual correction of the automated segmentations. Performance of the network was comparable between our clinical dataset and BRATS testing dataset when measured using the Dice coefficient. We further assessed the validity of our segmentation approach by evaluating the prognostic performance of volumetric features. Higher CER/TC and NC/TC were independently associated with higher risk of death overall. NET/WT was associated with lower risk of death in biopsy patients.

We demonstrate the possibility of transfer learning by segmenting tumour regions on a heterogeneous clinical dataset trained and tested on an independent dataset using multimodal imaging. A major advantage of our method is that it is applicable to data from different scanners and institutions. Furthermore, curating a large sample of uniform images for deep learning training is time- and labour-intensive [28]. Even when training time is considered, use of our computer-assisted segmentation method can reduce annotation time by 20 min per patient compared to fully manual segmentation. Training on an external dataset also increases the generalizability of the method and means data does not need to be split into training and validation cohorts, reducing sample sizes.

Deep learning methods can provide quantitative imaging-based prognostic biomarkers that outperform semi-quantitative estimates. In previous studies, tumour size has been investigated as a potential prognostic marker [29]. Tumour dimensions can be estimated using long axis diameter, cuboid, spheroid and ellipsoid formulas [30]. These methods are subjective and difficult to reproduce, leading to conflicting associations with survival [31, 32]. Formula-based estimates also have poor accordance with volumetric measurements [30].

We did not perform full manual segmentation as an approximate for ground truth to compare with our automated segmentations as the goal of our study was to determine if deep learning generated automated segmentations could be used to aid manual segmentation rather than as a replacement. Despite being trained on BRATS data, our network was able to detect the tumour in nearly all our patients and showed comparable segmentation accuracy in our dataset when evaluated against BRATS test data.

Our final segmentations were derived from corrections performed on the automated labels so it is expected that the label overlap for each subregion is significantly higher in our dataset compared to the BRATS data where no bias exists due to the manual segmentations being performed independently. Nonetheless, the relative accuracy of the segmentation labels across different tumour subregions was similar across the two test datasets. For example, the necrosis subregion had the highest proportion of misclassified voxels whilst the CER had the least proportion of misclassified voxels. This indicates that our manual corrections were dependent on the accuracy of the automated labels.

Few studies have compared accuracy of automated and manual segmentations of tumour subregions such as necrosis [15]. It is important to measure these subregions as they may have prognostic significance. Our data highlights the importance of choosing clinically relevant metrics to compare automated and manual segmentations. Two segmentations may have high Dice overlap but significantly different volumes if a smaller volume is entirely within the larger volume. There were significant differences between the automated and corrected segmentations for all tumour subregions apart from necrosis. Necrosis may be particularly challenging to segment using either automated or manual methods due to its heterogeneous and dispersed nature within the tumour core [15].

Necrosis was one of the earliest imaging markers found to have prognostic value [32]. Descriptive studies classifying tumours by estimating necrotic proportions have not been consistently prognostic [1]. In concordance with previous studies, we do not find a consistent association between necrosis volume measured by VASARI score and OS [33]. Instead, higher relative proportion of necrosis is associated with worse prognosis. NC/TC has been previously associated with worse survival [30, 34]. However, this study only included patients suitable for CRET, raising the possibility of selection bias. Furthermore, they did not control for qualitative imaging features described in the VASARI feature set in their multivariable analysis [34]. Hypoxia may select for quiescent stem-like cells around the NC which resist apoptosis and undergo proliferation [35].

Established prognostic variables including EOR calculated using our segmentations are associated with survival. The median OS in our cohort is only 9 months because compared to previous cohorts, we did not exclude patients undergoing biopsy [1, 30]. The median OS of 13 months in our resection patients is comparable to previous studies [1]. By including both resection and biopsy patients, our sample is representative of the heterogeneous cohorts of glioblastomas encountered in routine clinical practice. *IDH* mutation was not found to be associated with improved survival but this finding may be limited by the small number of *IDH*-mutant patients in our cohort. Our finding that *MGMT* methylation was not associated with improved survival is difficult to interpret. Not all studies demonstrate an association between *MGMT* methylation and improved OS, with several studies including a randomised control trial showing no association with survival [4, 36]. The reasons for this may be due to assay variability and difficulties correlating promoter methylation with protein expression [37].

The association between FLAIR proportion relative to other subregions and survival is unclear [38]. PTE/TC was not found to be associated with OS in a previous study [34]. Multiparametric biopsies have shown high levels of viable tumour cells within the non-enhancing region [39]. However, unlike our study, these studies did not differentiate oedema from non-enhancing tumour. The Dice coefficient between the automated and corrected FLAIR region segmentations was not significantly higher for our sample compared to BRATS as we were able to manually delineate NET from the FLAIR region.

Differentiating non-enhancing tumour from peritumoural oedema is important as each subregion may yield different prognostic information. The non-enhancing tumour within the FLAIR abnormality may represent lower grade disease [40]. We show that non-enhancing tumour proportion relative to the whole-tumour volume rather than FLAIR volume or peritumoural oedema volume was associated with improved survival in the biopsy group. This suggests that non-enhancing tumour was differentiated from peritumoural oedema by manual segmentation. Although our network was not able to detect non-enhancing tumour, we have shown high segmentation accuracy for the FLAIR region which can aid the manual delineation of the non-enhancing tumour.

Non-enhancing tumour variables were not prognostic in resection patients. This may be because the resection patients had significantly smaller volumes of non-enhancing tumour compared to the biopsy patients. The difficulty in delineating oedema from non-enhancing tumour may result in overlap between non-enhancing tumour and oedema in resection patients. We have shown that diffusion MRI signatures have higher sensitivity for invasive tumour can also be segmented using CNN [21, 41]. These biomarkers may be correlated with the FLAIR region to improve identification of infiltrative tumour.

CER/TC was associated with survival, independent of RTV and other prognostic factors. In radiogenomic studies, the CER correlates with genes involved in angiogenesis and hypoxia [42]. The relationship between the NC and the CER may not be linear; we show that NC/TC is independent of CER/TC and core tumour volume. This suggests that some tumours have relatively greater proportions of necrosis for a given tumour volume.

CER volume was negatively associated with survival in a cohort of resected glioblastomas but the accuracy of the volumetric measurements was limited by digitised hard-copy imaging [43]. CER volume has also been associated with worse survival when adjusted for other VASARI variables but in this study adjuvant treatment received was not controlled for [9]. We found that CER/TC rather than CER volume was significantly associated with survival. CER/TC may be a more accurate prognostic measure because it relates CER to the core tumour volume rather than whole-tumour volume as the oedema component may be affected by factors such as steroid use.

Limitations to our study include its retrospective nature. It is one of the largest studies investigating volumetric features and prognosis incorporating quantitative measurement of postoperative imaging and clinical variables. Future work to evaluate our approach should quantitatively compare segmentation measurements with manual segmentations from multiple observers to assess inter-rater variability [28]. In addition, an independent dataset is necessary for us to compare the relative prognostic performance of automated segmentations against manual segmentations and determine the reproducibility of our segmentations. Finally, volumetric features may be combined with texture- and shape-based analysis of tumour subregions to develop improved prognostic models for glioblastoma patients [44, 45].

## Conclusions

Using a CNN with a transfer learning approach, we have shown that volumetric measurements of glioblastoma tumour subregions can be measured from preoperative MRI with high accuracy. The CNN can be integrated into a radiological workflow to significantly shorten segmentation time.

Tumours with greater proportions of necrosis and contrast enhancement are independently associated with worse survival whilst non-enhancing tumour proportion is associated with improved survival. With further validation, we may be able to use volumetric features from routine clinical imaging for patient prognostication and stratification into clinical trials.

## Electronic supplementary material

ESM 1 (DOC 238 kb)

## Data Availability

Code can be made available by request to the authors

## Abbreviations

AIC: Akaike information criterion
5-ALA: 5-Aminoleuvenic acid
ASA: American Association of Anesthesiologists
BRATS: Multimodal brain tumour segmentation challenge
CER: Contrast-enhancing region
CRET: Complete resection of enhancing tumour
CNN: Convolutional neural network
EOR: Extent of resection
FOV: Field of view
FLAIR: Fluid attenuated inversion recovery
HR: Hazard ratio
IDH: Isocitrate dehydrogenase
KPS: Karnofsky Performance Scale
PTE: Peritumoural oedema
MGMT: O^6^-Methylguanine–DNA methyltransferase
MRI: Magnetic resonance imaging
NC: Necrotic core
NET: Non-enhancing tumour
OS: Overall survival
PACS: Picture archiving system
PRET: Partial resection of enhancing tumour
RTV: Residue tumour volume
SVZ: Subventricular zone
TC: Tumour core
VASARI: Visually Accessible Rembrandt Images
WT: Whole tumour
