# Supplementary material for: Deep learning for glioblastoma segmentation using preoperative magnetic resonance imaging identifies volumetric features associated with survival
Source: Acta Neurochir (Wien). 2020 Jul 13;162(12):3067–80. doi: 10.1007/s00701-020-04483-7 (PMC7593295; doi:10.1007/s00701-020-04483-7)
Supplement: Supplementary file 1 — (DOC 238 kb) [file 701_2020_4483_MOESM1_ESM.doc]

**Supplementary data**

*Abbreviations*

ASA = American association of anesthesiologists; CER = Contrast-enhancing region; CRET = Complete resection of enhancing tumour; EOR = Extent of resection; FLAIR = Fluid attenuated inversion recovery; KPS = Karnofsky performance status; IDH = Isocitrate Dehydrogenase; MGMT- O^6^-methylguanine DNA methyltransferase; NS = Not significant; NC = Necrotic core; NER = Non-enhancing region; NET = Non-enhancing tumour; PRET = Partial resection of enhancing tumour; PTE/NET = Peritumoural edema; RTV = Residual tumour volume; SVZ = Subventricular zone; TC = Tumour core; VASARI = Visually AcceSAble Rembrandt Images; WT = Whole tumour.

A: Methods

*Definitions for manual segmentations*

1. Necrotic core (NC): hypointense region on T1-weighted imaging that is usually surrounded by a rim of enhancing region on T1C images.
2. Contrast enhancing region (CER): enhancing region on T1C surrounding the hypointense necrotic centre, excluding any vessels in the peri-tumoural region.
3. Non-enhancing tumour (NET): hyperintense heterogenous region on T2-weighted imaging which does not enhance on T1C. It is more heterogenous and has a lower signal intensity than vasogenic oedema on T2-weighted images.
4. Peritumoural oedema (PTE): hyperintense region on T2-weighted imaging and hypointense region on T1-weighted images which predominantly involve the white-matter and generally respect the WM/grey-matter boundaries.
5. Whole tumour (WT): CER + NC + NET + PTE.
6. Tumour core (TC): CER + NC + NET.
7. Fluid attenuated inversion recovery (FLAIR) region (NER): PTE + NET.

*Imaging characteristics*

Preoperative imaging was acquired in a 1.5 Tesla (1.5-T) MRI system or a 3-T MRI system (GE Healthcare). T1-weighted images were acquired before and following intravenous injection of 9 ml of gadolinium (Gadovist, Bayer Schering Pharma). Some T1-weighted FLAIR sequences were acquired (n = 7). The Field of View (FOV) was 256 x 256 with 142 – 252 slices apart from one patient with a FOV of 512 x 512 x 23 (voxel size: 0.5 mm x 0.5 mm x 7 mm). The remaining patients had isotropic 1 mm^3^ voxel size.

Postoperative imaging was acquired 72-h following surgery from the same 1.5-T MRI system. 2D T1, T1C and 2D FLAIR images were acquired (FOV: 512 x 512 x 21 – 23).

A1. Parameters of preoperative and postoperative MRI sequences.

|  | T1/FLAIR | T1C | T2 FLAIR | T2 |
| --- | --- | --- | --- | --- |
| Sequence | SE | GE/IR | SE/IR | SE |
| Preoperative: 1.5-T Optima 450W/3-T Optima 750W MRI systems | | | | |
| TR | 540/2589 | 8 | 8000 – 9304 | 5403 – 7318 |
| TE | 12/24 | 3 | 125 – 159 | 81 – 100 |
| Inversion | NA/806 | 450 – 900 | 2129 – 2383 | N/A |
| Thickness | 6 | 1 | 6 | 6 |
| Flip angle | 90/111 | 9 – 12 | 111 – 160 | 111 – 160 |
| Postoperative: 3-T Optima 450W MRI system | | | | |
| TR | 500 | 400 | 8492 | 6822 |
| TE | 12 | 20 | 160 | 101 |
| Inversion | NA | NA | 2290 | N/A |
| Thickness | 6 | 6 | 6 | 6 |
| Flip angle | 90 | 90 | 160 | 160 |

FLAIR = Fluid-attenuated inversion recovery; SE = spin echo; GE/IR = gradient echo/inverse recovery; EP = echo planar; TR = repetition time (mmsec); TE = echo time (mmsec); Flip angle (**°**); NA = not applicable.

**Table A2**. VASARI features scored on preoperative MRI

| Feature | Scoring | Description |
| --- | --- | --- |
| Tumour location | Frontal, temporal, insular, parietal, occipital, corpus callosum, brainstem | Location based on epicentre of CER or NER |
| Side | Right, bilateral/center, left | Side of epicentre |
| Eloquence | Speech motor, speech receptive, motor, vision | Any CER/NER involvement |
| Enhancement | None, minimal, avid | Degree of enhancement on T1C compared to T1 |
| Enhancing proportion | < 5%, 6 – 33%, 34 - 67%, 68 - 95% | Estimated proportion compared to other tumour subregions |
| Non-enhancing proportion | < 5%, 6 – 33%, 34 - 67%, 68 - 95% < 95%, 100% | Estimated proportion compared to other tumour subregions |
| Necrosis proportion | None, < 5%, 6 – 33%, 34 - 67%, | Estimated proportion compared to other tumour subregions |
| Oedema proportion | None, < 5%, 6 – 33%, 34 - 67%, | Estimated proportion compared to other tumour subregions |
| Multifocal/multicentric | Multifocal, multicentric, gliomatosis | Multifocal- at least one tumour region not contagious with dominant lesion, multicentric- lesions in separate lobes, gliomatosis- generalised involvement of WM |
| Ependymal extension | No, yes | Enhancement of ependymal surface touching CER/NER |
| Cortical involvement | No, yes | Can cortex be distinguished from tumour |
| Deep white matter involvement | No, internal capsule, brainstem, corpus callosum | CER/NER extending into deep white matter |
| NER crossing midline | No, yes | NER crossing into contralateral hemisphere |
| CER crossing midline | No, yes | CER crossing into contralateral hemisphere |

B: Baseline characteristics of cohort

**Table B1. Baseline characteristics of patients comparing surgical groups**.

| Characteristics | Biopsy  (n = 45) | Resection  (n = 75) | *p* |
| --- | --- | --- | --- |
| Age [IQR]*^a^* | 67.6  [62.4 – 72.6] | 63.4  [53.6 – 68.4] | 0.01 |
| Male (%) | 24 (53.3) | 45 (60.0) | NS |
| KPS (%) | |  | NS |
| <70 | 1 (2.2) | 1 (1.3) | NS |
| 70 | 3 (6.7) | 2 (2.7) | NS |
| 80 | 15 (33.3) | 5 (6.7) | < 0.001 |
| 90 | 19 (42.2) | 41 (54.7) | NS |
| 100 | 7 (15.6) | 26 (34.7) | 0.02 |
| Presenting symptoms (%) | |  |  |
| Headache | 14 (31.1) | 30 (40.0) | NS |
| Seizure | 16 (35.6) | 22 (29.3) | NS |
| Cognitive | 20 (44.4) | 31 (41.3) | NS |
| Vision | 4 (8.9) | 20 (26.7) | 0.02 |
| Language | 21 (46.7) | 24 (32.0) | NS |
| Motor | 18 (40.0) | 28 (37.3) | NS |
| Sensory | 12 (26.7) | 6 (8.0) | 0.006 |
| Perioperative variables (%) | |  |  |
| Steroid use | 37 (82.2) | 71 (94.7) | 0.03 |
| ASA grade |  |  |  |
| 1 | 0 (0) | 8 (10.7) | 0.02 |
| 2 | 33 (73.3) | 53 (70.7) | NS |
| 3 | 12 (26.7) | 13 (17.3) | NS |
| 4 | 0 (0) | 1 (1.3) | NS |
| Preoperative deficit | 45 (100) | 72 (96.0) | NS |
| Postoperative deficit | |  |  |
| None | 21 (46.7) | 33 (44.0) | NS |
| New/worsened | 12 (26.7) | 7 (22.7) | NS |
| Improved | 12 (26.7) | 25 (33.3) | NS |
| Tumour variables (%) | |  |  |
| *IDH*-mutant | 0 (0) | 4 (5.3) | NS |
| *MGMT*-methylated | 45 (40.0) | 71 (45.3) | NS |
| Adjuvant therapy (%) | |  |  |
| Chemoradiotherapy | 20 (44.4) | 56 (74.7) | 0.001 |
| Radiotherapy | 16 (35.6) | 11 (14.7) | 0.008 |
| None | 9 (20.0) | 8 (10.7) | NS |
| Median overall survival*^b^*  (months) (95% CI) | 4.7  (2.7 – 8.6) | 12.8  (7.5 – 16.8) | <0.001 |
| Alive at last follow-up (%) | 4 (16.0) | 21 (84.0) | 0.01 |
| Volumetric variables | | | |
| WT volume | 75.3 (36.5 – 114.6) | 79.7  (45.9 – 116.6) | NS |
| TC volume | 25.7 (16.2 – 44.3) | 25.2  (15.2 – 41.9) | NS |
| PTE volume | 37.3 (23.3 – 65.3) | 47.3  (27.1 – 74.5) | NS |
| CER volume | 5.4 (1.4 – 10.6) | 10.3  (4.8 – 17.5) | 0.002 |
| NET volume | 2.6 (0 – 20.9) | 0.4 (0 – 4.5) | 0.003 |
| NC volume | 3.6 (0.8 – 13.8) | 8.6 (4.3 – 19.2) | 0.015 |

CER = Contrast-enhancing region; KPS = Karnofsky performance scale; ASA = American Association of Anesthesiologists; GTR = Gross Total Resection; STR = Subtotal Resection; RTV = Residual Tumour Volume; IQR = Interquartile Range; IDH = Isocitrate Dehydrogenase; MGMT = O^6^-methylguanine–DNA methyltransferase. NC = Necrotic core; NS = Not significant. PTE = Peritumoural edema; TC = Tumour core; WT = Whole tumour. *^a^*Median [Interquartile range]. *^b^*Log-rank test of equality of survivor function.

**Table B2**. Baseline imaging characteristics of preoperative MRI. (n = 120)

| Characteristics | All patients | Biopsy  (n = 45) | Resection  (n = 75) | *p* |
| --- | --- | --- | --- | --- |
| VASARI variables (%) | |  |  |  |
| Left side | 71 (59.2) | 31 (68.9) | 40 (53.3) | NS |
| Bilateral | 1 (0.8) | 1 (0) | 0 (0) | NS |
| Location |  |  |  |  |
| Frontal | 35 (29.2) | 11 (24.4) | 24 (32.0) | NS |
| Temporal | 49 (40.8) | 15 (33.3) | 34 (45.3) | NS |
| Insula | 3 (2.5) | 3 (6.7) | 0 (0) | NS |
| Parietal | 24 (20.0) | 10 (22.2) | 14 (18.7) | NS |
| Occipital | 4 (3.3) | 1 (2.2) | 3 (4.0) | NS |
| Brainstem | 1 (0.8) | 1 (2.2) | 0 (0) | NS |
| Callosum | 4 (3.3) | 4 (8.9) | 0 (0) | NS |
| Eloquence |  |  |  |  |
| None | 60 (50.0) | 10 (22.2) | 50 (66.7) | < 0.001 |
| Speech motor | 12 (10.0) | 4 (8.9) | 8 (10.7) | NS |
| Speech receptive | 13 (10.8) | 7 (15.6) | 6 (8.0) | NS |
| Motor | 17 (14.2) | 9 (20.0) | 8 (10.7) | NS |
| Vision | 18 (15.0) | 15 (33.3) | 3 (4.0) | 0.02 |
| SVZ involvement | 55 (45.8) | 37 (82.2) | 18 (24.0) | < 0.001 |
| Cortical involvement | 115 (95.8) | 42 (93.3) | 73 (97.3) | NS |
| White matter involvement | 41 (34.2) | 32 (71.1) | 9 (12.0) | < 0.001 |
| Multifocal | 15 (12.5) | 10 (22.2) | 5 (6.7) | 0.01 |
| NER crossing | 13 (10.8) | 10 (76.9) | 3 (23.1) | 0.002 |
| CER crossing | 8 (6.7) | 7 (87.5) | 1 (12.5) | 0.003 |
| Proportion PTE | |  |  |  |
| 0% | 10 (8.4) | 3 (6.7) | 7 (9.5) | NS |
| <5% | 56 (47.1) | 23 (51.1) | 33 (44.6) | NS |
| 6 – 33% | 38 (31.9) | 4 (31.1) | 24 (32.4) | NS |
| 34 – 67% | 15 (12.6) | 5 (11.1) | 10 (13.5) | NS |
| Proportion CER | |  |  |  |
| <5% | 46 (38.3) | 16 (35.6) | 30 (40.0) | NS |
| 6 – 33% | 49 (40.8) | 9 (42.2) | 30 (40.0) | NS |
| 34 – 67% | 18 (15.0) | 7 (15.6) | 11 (14.7) | NS |
| 68 – 95% | 7 (5.8) | 3 (6.7) | 4 (5.3) | NS |
| Proportion NER | |  |  |  |
| <5% | 38 (31.7) | 15 (33.3) | 3 (30.7) | NS |
| 6 – 33% | 33 (27.5) | 12 (26.7) | 21 (28.0) | NS |
| 34 – 67% | 28 (23.3) | 10 (22.2) | 18 (24.0) | NS |
| 68 – 95% | 13 (10.8) | 6 (13.3) | 7 (9.3) | NS |
| <95% | 7 (5.8) | 1 (2.2) | 6 (8.0) | NS |
| 100% | 1 (0.8) | 1 (2.2) | 0 (0) | NS |
| Proportion NC | |  |  |  |
| 0% | 4 (3.3) | 2 (2.7) | 2 (4.4) | NS |
| <5% | 36 (30.0) | 22 (48.9) | 14 (18.7) | <0.001 |
| 6 – 33% | 48 (40.0) | 12 (26.7) | 36 (48.0) | <0.020 |
| 34 – 67% | 32 (26.7) | 9 (20.0) | 23 (30.7) | NS |
| Volumetric variables*^a^* | | | | |
| WT volume | 77.0 (43.6 – 115.6) | 75.3 (36.5 – 114.6) | 79.7 (45.9 – 116.6) | NS |
| TC volume | 25.4 (15.4 – 44.0) | 25.7 (16.2 – 44.3) | 25.2 (15.2 – 41.9) | NS |
| PTE volume | 41.6 (26.0 – 68.7) | 37.3 (23.3 – 65.3) | 47.3 (22.1 – 74.5) | NS |
| CER volume | 8.2 (2.9 – 14.9) | 5.4 (1.4 – 10.6) | 10.3 (4.8 – 17.5) | 0.002 |
| NET volume | 0.7 (0 – 7.5) | 2.6 (0 – 20.9) | 0.4 (0 – 4.5) | 0.03 |
| NC volume | 7.2 (2.7 – 16.2) | 3.6 (0.8 – 13.8) | 8.6 (4.3 – 19.2) | 0.02 |
| FLAIR/WT ratio | 0.7 (0.6 – 0.9) | 0.7 (0.6 – 0.9) | 0.7 (0.6 – 0.8) | NS |
| PTE/WT ratio | 0.64 (0.49 – 0.76) | 0.57  (0.37 – 0.72) | 0.65  (0.51 - 0.77) | NS |
| NET/WT ratio | 0.01 (0 – 0.13) | 0.03 (0 – 0.32) | 0.00 (0 – 0.06) | 0.04 |
| CER/WT ratio | 0.12 (0.05 – 0.20) | 0.10  (0.08 – 0.21) | 0.08  (0.08 – 0.21) | NS |
| NC/TC ratio | 0.4 (0.2 – 0.5) | 0.3  (0.04 – 0.57) | 0.4 (0.3 – 0.5) | 0.02 |
| NET/TC ratio | 0.03 (0 – 0.3) | 0.1 (0 – 0.8) | 0.19 (0 – 0.20) | 0.03 |
| CER/TC ratio | 0.5 (0.2 – 0.6) | 0.3  (0.04 – 0.57) | 0.5 (0.2 – 0.6) | 0.05 |
| PTE/TC ratio | 1.8 (1.0 – 3.1) | 1.4 (0.6 – 2.5) | 1.8 (1.0 – 3.4) | NS |
| PTE/CER ratio | 5.4 (2.5 – 10.7) | 5.7 (3.2 – 17.6) | 5.3 (2.3 – 9.2) | 0.05 |
| PTE/NET ratio | 5.9 (1.6 – 17.3) | 2.6 (0.7 – 7.2) | 10.8 (2.2 – 22.0) | 0.01 |
| PTE/NC ratio | 5.3 (2.4 – 13.0) | 5.0 (1.9 – 19.4) | 5.4 (2.8 – 12.0) | NS |

*^a^*Median (cm^3^) (IQR). CER = Contrast-enhancing region; KPS = Karnofsky performance scale; ASA = American Association of Anesthesiologists; GTR = Gross Total Resection; STR = Subtotal Resection; RTV = Residual Tumour Volume; IQR = Interquartile Range; IDH = Isocitrate Dehydrogenase; MGMT = O^6^-methylguanine–DNA methyltransferase. NC = Necrotic core; NS = Not significant. PTE = Peritumoural edema; TC = Tumour core; WT = Whole tumour.

C: Cox regression models

Bivariable cox regression for OS was performed for all patients, resection patient and biopsy patient groups. Multivariable cox regression models included known prognostic variables including age, KPS and any predictor variable which was significant (*p* < 0.2) in bivariable regression models.

**Table C1. Correlations between significant volumetric variables for all patients**.

| Variable | FLAIR volume | CER/WT ratio | CER/TC ratio | NC/TC ratio | PTE/NET ratio |
| --- | --- | --- | --- | --- | --- |
| FLAIR volume | - | -0.159 | 0.017 | 0.178 | 0.509 |
|  | - | NS | NS | NS | <0.001 |
| CER/WT ratio | -0.159 | - | 0.794 | -0.079 | 0.402 |
|  | NS | - | < 0.001 | NS | < 0.001 |
| CER/TC ratio | 0.017 | 0.794 | - | -0.161 | 0.661 |
|  | NS | < 0.001 | - | NS | < 0.001 |
| NC/TC ratio | 0.178 | -0.079 | -0.161 | - | 0.607 |
|  | NS | NS | NS | - | < 0.001 |
| PTE/NET ratio | 0.509 | 0.402 | 0.661 | 0.607 | - |
|  | <0.001 | < 0.001 | < 0.001 | < 0.001 | - |

CER = Contrast-enhancing region; FLAIR = Fluid attenuated inversion recovery; NET = Non-enhancing tumour; NC = Necrotic core; NS = Not significant. PTE = Peritumoural edema; TC = Tumour core; WT = Whole tumour.

**Table C2**. Akaike Information Criterion (AIC) values for each model for all patients (n = 120).

|  | Model | AIC |
| --- | --- | --- |
| 1 | FLAIR volume + CER/WT ratio | 727.02 |
| 2 | FLAIR volume + CER/TC ratio | 725.05 |
| 3 | FLAIR volume + NC/TC ratio | 724.98 |
| 4 | CER/WT ratio + NC/TC ratio | 718.30 |
| **5** | **CER/TC ratio + NC/TC ratio** | **717.20** |
| 6 | FLAIR volume + NC/TC ratio + CER/WT ratio | 720.30 |
| 7 | FLAIR volume + NC/TC ratio + CER/TC ratio | 718.06 |

CER = Contrast-enhancing region; FLAIR = Fluid attenuated inversion recovery; NC = Necrotic core; NS = Not significant. PTE = Peritumoural edema; TC = Tumour core; WT = Whole tumour.

**Table C3. Correlations between significant volumetric variables for biopsy patients.**

| Variable | NET  volume | NC volume | NET/TC ratio | CER/TC ratio | NC/TC ratio | FLAIR/WT ratio | NET/WT ratio | NC/WT ratio | PTE/NET ratio |
| --- | --- | --- | --- | --- | --- | --- | --- | --- | --- |
| NET  volume | - | -0.194 | 1.000 | -0.750 | -0.502 | 0.643 | 0.944 | -0.345 | -0.705 |
|  | - | NS | <0.001 | <0.001 | 0.004 | <0.001 | <0.001 | 0.0205 | <0.001 |
| NC volume | -0.194 | - | -0.315 | -0.035 | 0.849 | -0.553 | -0.206 | 0.915 | 0.424 |
|  | NS | - | 0.035 | NS | <0.001 | 0.001 | NS | <0.001 | 0.027 |
| NET/TC ratio | 1.000 | -0.315 | - | -0.796 | -0.569 | -0.266 | 0.961 | -0.422 | -0.782 |
|  | <0.001 | 0.035 | - | <0.001 | <0.001 | NS | <0.001 | 0.004 | <0.001 |
| CER/TC ratio | -0.750 | -0.035 | -0.796 | - | 0.128 | 0.317 | -0.778 | 0.018 | 0.669 |
|  | <0.001 | NS | <0.001 | - | NS | 0.033 | <0.001 | NS | 0.001 |
| NC/TC ratio | -0.502 | 0.849 | -0.569 | 0.128 | - | 0.156 | -0.498 | 0.906 | 0.584 |
|  | 0.004 | <0.001 | <0.001 | NS | - | NS | 0.005 | <0.001 | 0.001 |
| FLAIR/WT ratio | 0.643 | -0.553 | -0.266 | 0.317 | 0.156 | - | 0.615 | -0.665 | -0.504 |
|  | <0.001 | 0.001 | NS | 0.033 | NS | - | <0.001 | <0.001 | 0.007 |
| NET/WT ratio | 0.944 | -0.206 | 0.961 | -0.778 | -0.498 | 0.615 | - | -0.292 | -0.988 |
|  | <0.001 | NS | <0.001 | <0.001 | 0.005 | <0.001 | - | NS | <0.001 |
| NC/WT ratio | -0.345 | 0.915 | -0.422 | 0.018 | 0.906 | -0.665 | -0.292 | - | 0.323 |
|  | 0.0205 | <0.001 | 0.004 | NS | <0.001 | <0.001 | NS | - | NS |
| PTE/NET ratio | -0.705 | 0.424 | -0.782 | 0.669 | 0.584 | -0.504 | -0.988 | 0.323 | - |
|  | <0.001 | 0.027 | <0.001 | 0.001 | 0.001 | 0.007 | <0.001 | NS | - |

CER = Contrast-enhancing region; FLAIR = Fluid attenuated inversion recovery; NET = Non-enhancing tumour; NC = Necrotic core; NS = Not significant. PTE = Peritumoural edema; TC = Tumour core; WT = Whole tumour.

**Table C4. Akaike Information Criterion** of multivariable models for biopsy patients.

|  | Model | AIC |
| --- | --- | --- |
| 1 | NET volume + NC volume | 230.10 |
| 2 | NC volume + CER/TC ratio | 230.81 |
| 3 | NC volume + NET/WT ratio | 226.56 |
| 4 | NET/TC ratio + FLAIR/WT ratio | 228.78 |
| 5 | CER/TC ratio + NC/TC ratio | 228.82 |
| 6 | CER/TC ratio + NC/WT ratio | 228.73 |
| 7 | NC/TC ratio + FLAIR/WT ratio | 232.09 |
| **8** | **NET/WT ratio + NC/WT ratio** | **226.54** |
| 9 | NC/WT ratio + PTE/NET ratio | - |

CER = Contrast-enhancing region; FLAIR = Fluid attenuated inversion recovery; NC = Necrotic core; NS = Not significant. PTE = Peritumoural edema; TC = Tumour core; WT = Whole tumour.

**Table C5**. Correlations between significant volumetric variables for resection patients.

| Variable | CER  volume | FLAIR/TC ratio | CER/TC ratio | PTE/TC  ratio | NC/TC ratio | CER/WT ratio | PTE/NC ratio |
| --- | --- | --- | --- | --- | --- | --- | --- |
| CER  volume | - | -0.321 | 0.504 | -0.228 | -0.081 | 0.663 | -0.207 |
|  | - | 0.005 | <0.001 | 0.050 | NS | <0.001 | NS |
| FLAIR/TC ratio | -0.321 | - | 0.081 | 0.973 | 0.034 | -0.531 | 0.754 |
|  | 0.005 | - | NS | <0.001 | NS | <0.001 | <0.001 |
| CER/TC ratio | 0.504 | 0.081 | - | 0.202 | -0.419 | 0.731 | 0.298 |
|  | <0.001 | NS | - | NS | 0.002 | <0.001 | 0.010 |
| PTE/TC ratio | -0.228 | 0.973 | 0.202 | - | 0.136 | -0.410 | 0.672 |
|  | 0.050 | <0.001 | NS | - | 0.243 | 0.003 | <0.001 |
| NC/TC  Ratio | -0.081 | 0.034 | -0.419 | 0.136 | - | -0.315 | -0.533 |
|  | NS | NS | 0.002 | 0.243 | - | 0.006 | <0.001 |
| CER/WT ratio | 0.663 | -0.531 | 0.731 | -0.410 | -0.315 | - | -0.263 |
|  | <0.001 | <0.001 | <0.001 | 0.003 | 0.006 | - | 0.024 |
| PTE/NC ratio | -0.207 | 0.754 | 0.298 | 0.672 | -0.533 | -0.263 | - |
|  | NS | <0.001 | 0.010 | <0.001 | <0.001 | 0.024 | - |

CER = Contrast-enhancing region; FLAIR = Fluid attenuated inversion recovery; NC = Necrotic core; NS = Not significant. PTE = Peritumoural edema; TC = Tumour core; WT = Whole tumour.

**Table C6**. Akaike Information Criterion of multivariable models for resection patients.

|  | Model | AIC |
| --- | --- | --- |
| 1 | CER volume + NC/TC ratio | 371.12 |
| **2** | **CER volume + PTE/NC ratio** | **351.10** |
| 3 | FLAIR/TC ratio + CER/TC ratio | 371.25 |
| 4 | FLAIR/TC ratio + NC/TC ratio | 371.98 |
| 5 | CER/TC ratio + PTE/TC ratio | 371.24 |
| 6 | CER/WT ratio | 370.08 |

CER = Contrast-enhancing region; FLAIR = Fluid attenuated inversion recovery; NC = Necrotic core; NS = Not significant. PTE = Peritumoural edema; TC = Tumour core; WT = Whole tumour.
